# Individual and area level factors associated with the breast cancer diagnostic-treatment interval in Queensland, Australia

**DOI:** 10.1007/s10549-023-07134-4

**Published:** 2023-11-06

**Authors:** James D. Retell, Jessica K. Cameron, Joanne F. Aitken, Philippa Youl, Chris Pyke, Jeff Dunn, Suzanne Chambers, Peter D. Baade

**Affiliations:** 1 Viertel Cancer Research Centre, Cancer Council Queensland, Brisbane, QLD Australia; 2 School of Public Health, University of Queensland, Brisbane, QLD Australia; 3 School of Public Health and Social Work, Queensland University of Technology, Brisbane, QLD Australia; 4 Institute for Resilient Regions, University of Southern Queensland, Brisbane, QLD Australia; 5 Cancer Alliance Queensland, Metro South Hospital and Health Service, Woolloongabba, QLD Australia; 6 Menzies Health Institute Queensland, Griffith University, Southport, QLD Australia; 7 School of Mathematical Sciences, Queensland University of Technology, Brisbane, QLD Australia; 8 Mater Hospital, Brisbane, QLD Australia; 9 Prostate Cancer Foundation of Australia, Sydney, NSW Australia; 10 Faculty of Health Sciences, Australian Catholic University, Sydney, NSW Australia

**Keywords:** Breast cancer, Delay, Diagnosis, Treatment, Inequalities, Australia, Health care

## Abstract

**Background:**

Delays to breast cancer treatment can lead to more aggressive and extensive treatments, increased expenses, increased psychological distress, and poorer survival. We explored the individual and area level factors associated with the interval between diagnosis and first treatment in a population-based cohort in Queensland, Australia.

**Methods:**

Data from 3216 Queensland women aged 20 to 79, diagnosed with invasive breast cancer (ICD-O-3 C50) between March 2010 and June 2013 were analysed. Diagnostic dates were sourced from the Queensland Cancer Registry and treatment dates were collected via self-report. Diagnostics-treatment intervals were modelled using flexible parametric survival methods.

**Results:**

The median interval between breast cancer diagnosis and first treatment was 15 days, with an interquartile range of 9–26 days. Longer diagnostic-treatment intervals were associated with a lack of private health coverage, lower pre-diagnostic income, first treatments other than breast conserving surgery, and residence outside a major city. The model explained a modest 13.7% of the variance in the diagnostic-treatment interval $\left( {R_{D}^{2} } \right)$. Sauerbrei’s *D* was 0.82, demonstrating low to moderate discrimination performance.

**Conclusion:**

Whilst this study identified several individual- and area-level factors associated with the time between breast cancer diagnosis and first treatment, much of the variation remained unexplained. Increased socioeconomic disadvantage appears to predict longer diagnostic-treatment intervals. Though some of the differences are small, many of the same factors have also been linked to screening and diagnostic delay. Given the potential for accumulation of delay at multiple stages along the diagnostic and treatment pathway, identifying and applying effective strategies address barriers to timely health care faced by socioeconomically disadvantaged women remains a priority.

**Supplementary Information:**

The online version contains supplementary material available at 10.1007/s10549-023-07134-4.

## Introduction

In 2020 breast cancer surpassed lung cancer as the most diagnosed cancer worldwide, with an estimated 2.3 million new cases [1]. Amongst women, breast cancer accounts for approximately 1 in 4 cancer diagnoses and 1 in 6 cancer-related deaths globally [1]. In Australia, the age-standardised incidence rate of breast cancer has been steadily increasing, and it was estimated that approximately 20,000 Australian women were diagnosed with breast cancer in 2021 [2]. Understanding the factors that contribute to and exacerbate the breast cancer burden remains a high priority.

In Australia, 5-year survival varies from as high as 99% for stage I breast cancer to 20–35% for stage IV breast cancer [2]. Critically, prolonged pathways to treatment are associated with larger tumour sizes, the presence of cancer cells in the lymph nodes, later stage cancers [3–9], and reduced survival [8, 10–12]. Moreover, increased wait-times for treatment can result in the need for more aggressive and extensive treatments [13], increased expenses and increased psychological distress [14]. At present, timely detection and treatment offer the best chance of improving breast cancer outcomes, and, consequently, are a key focus of breast cancer management and care [15]. However, improving timely care and treatment first requires identifying the factors that contribute to unnecessary delay.

There is an extensive literature examining time to *detection* and *diagnosis* [16], with socioeconomic disadvantage typically associated with later detection and longer wait times for diagnosis [15, 17–19]. However, the interval between diagnosis and treatment has received relatively less attention in the literature. Studies carried out in various countries suggest that many of the factors associated with later detection and diagnosis also predict the length of the diagnosis-treatment interval, for example, ethnicity/race [20, 21], education [13], and remoteness [22]. However, we are aware of no large-scale studies investigating the extent to which individual and area level factors affect the time interval between diagnosis and treatment in an Australian context. Given that accumulation of delay at multiple stages along the diagnostic and treatment pathway may harm outcomes and contribute to the breast cancer outcome gradients seen in Australia [19, 23], it is important to identify factors that potentially may affect the wait-time for initial treatment following a breast cancer diagnosis. As such, we analysed data from a large population-based study of women diagnosed with breast cancer in Queensland, Australia to quantify the wait time between diagnosis and *first* treatment and to identify any individual and area level factors that predict the interval.

## Methods

### Study population

Analyses were conducted using data from the Breast Cancer Outcome Study—a longitudinal study of women from Queensland, Australia aged 20 to 79 years with a histologically confirmed diagnosis of invasive breast cancer between the 1st of March 2010 and the 30th of June 2013. The study used telephone and self-administered questionnaires to collect individual-level data from English speaking women diagnosed with invasive breast cancer, identified through the Queensland Cancer Register. Clinical, diagnostic, and treatment information was obtained from medical records at 12 months post-diagnosis. A total of 5426 potentially eligible women were identified through the Queensland Cancer Register, of whom 3326 (61%) met all eligibility criteria and responded. Full details of the eligibility criteria, data sourcing, and the telephone interview are described in [23]. Women living in major cities were less likely to participate (*p* = 0.04), and *non-respondents* were more likely to be diagnosed with advanced disease (*p* = 0.03) [23].

### Data preparation

Date of breast cancer diagnosis and clinical data was sourced from the Queensland Cancer Register, where diagnostic date reflects the first date of investigation from pathology, or the date indicated on hospital admission notification. First treatment date and type were collected through self-report. Patient self-reported date of surgeries (94% of the breast conserving surgery, 87% of the mastectomy) was cross checked with clinical records and the correlation was high (*r* = 0.99), indicating a high degree of reliability for the 95.1% of women whose first treatment was surgical. The diagnostic-treatment interval was modelled as the number of days between diagnosis and first treatment. Specifically, we modelled diagnostic-treatment intervals up to a maximum of 3 months (90 days). Thirteen cases with intervals greater than 90 days were treated as censored. Left uncensored, these extreme observations increased model complexity, in the form are greater degrees of freedom and the need for additional time-dependencies, and, in some cases, caused model convergence issues. Importantly, censoring these extreme values had minimal effect on the coefficient estimates and model fit. Ninety-seven respondents reported their first treatment occurring prior to the date of diagnosis. That is, they had a *negative* diagnostic-treatment interval. Many of these intervals, if not most, can be assumed to reflect data entry errors given the median length of the negative diagnostic-treatment intervals was − 168 days. As the negative intervals cannot be modelled here, all negative intervals were removed. Negative diagnostic-treatment intervals were associated with later stage breast cancer at diagnosis (*p* = 0.04) and first treatments *other than* BCS or mastectomy (*p* < 0.001). Separately, 123 participants reported inconsistent information with respect to first treatment type and first treatment date and were removed. These discrepant cases overlapped greatly (58%) with negative diagnostic-treatment intervals (*p* < 0.001) and were strongly associated with first treatment types other than BCS and mastectomy (*p* < 0.001).

One-hundred and eighty-seven women reported the same date for diagnosis and first-treatment—an interval of zero days. The majority of these intervals likely reflect confirmation of diagnosis at treatment and were recoded as 0.5 to allow for the modelling of log-time. Importantly, inspection of interval data revealed a severely compressed distribution—many ties concentrated below the median. To break ties and resolve model convergence issues that arose with increasing model complexity, we applied uncorrelated uniform noise—$Unif\left( {0, \pm\,0.01} \right)$ days—to the diagnostic-treatment interval. To discount the introduction of bias, we compared coefficient estimates derived from the original interval data to those derived from the transmuted (plus noise) data, using simplified Weibull (df = 1) models. Only negligible differences were observed that did not affect interpretation.

Finally, all treatment types other than breast conserving surgery (BCS) or mastectomy (chemotherapy, Herceptin, hormone, radiotherapy, other surgery, or therapy) were aggregated as ‘other’ due to small counts in each of these categories. Remoteness of residence when diagnosed with breast cancer was categorised using the Australian Bureau of Statistics Remoteness Index (ARIA+), which is a measure of accessibility and remoteness based on geographical location [24]. We aggregated the Outer Regional, Remote and Very Remote categories due to small counts in each of these categories. Age at diagnosis was mean centred and visual inspection of martingale residuals showed that age at diagnosis appeared approximately linear on the log cumulative hazard scale.

### Model derivation

The length of the diagnostic-treatment interval (days) was modelled using flexible parametric survival analysis (Royston–Parmar models). The approach involves fitting restricted cubic polynomial splines to flexibly model the baseline log-cumulative hazard. The method allows the estimation of absolute measures of effect (e.g. baseline hazard rates) at all time points, and extrapolation of the time-to-event function. Additionally, the use of splines addresses the potentially unrealistic assumption of constant or monotonic hazard inherent to parametric survival models [25]. The selection of scales and number of degrees of freedom (knots) for the baseline spline function was made using the Bayesian information criterion (BIC) statistic and Akaike information criterion (AIC). Knot position was set using 2 boundary knots (smallest and largest uncensored log interval-times) and *m* interior knots based on empirical centiles of the log treatment-time distribution [26]. The data were best captured using the log cumulative hazard (proportional hazard) scale with 3 degrees of freedom—log cumulative odds models with degrees of freedom > 2 failed to converge.

Candidate predictors and potential confounders were identified from a literature search of individual and area level factors previously associated with delays to cancer detection, diagnosis and treatment, and cancer outcomes more broadly. Potential model covariates were identified using univariable analyses coupled with likelihood ratio tests. Covariates with evidence of an association with the diagnostic-treatment interval at *p* < 0.3 were tested in the multivariable building process. We then used an iterative backwards selection process and removed predictors one at time to arrive at the final adjusted multivariable model. To test for time-dependent effects (TD), we first entered a time-dependent effect for each covariate into the multivariable model separately. We then used a forward selection process, entering time-dependent terms into the model one at a time, in order of greatest evidence of time-dependency (lowest *p* value). Time dependencies were identified using likelihood-ratio tests and were only included in the final model when *p* < 0.01. A more conservative threshold was used to buffer against over-fitting and multiple testing. For simplicity, time-dependent effects were initially modelled using the same degrees of freedom as the baseline spline function (df = 3). Once appropriate time-dependent effects had been identified, BIC and AIC criteria indicated that a solution with 2 degrees of freedom for time-dependent effects provided the best model fit.

In contrast to typical applications of survival analysis, here we are modelling time to *treatment*—a desirable outcome—so the interpretation of model coefficients is in a sense reversed. Specifically, coefficients *less* than 1 convey a disadvantage—less likely to have received treatment by a given timepoint. For clarity, where one might otherwise refer to survival curves, here we refer to *treatment* curves, and their shape is the inverse to traditional survival curves. Additionally, hazard ratios are referred to as treatment ratios. Analyses were run in Stata (version 16.1, StataCorp) using the stpm2 package.

### Model discrimination

The discrimination performance reflects the ability of a time-to-event model to assign higher risks to individuals who experience earlier events—those who indeed have higher risk of the event. We assessed the discrimination performance of our model using Royston and Sauberbrei’s *D* statistic. Royston and Sauberbrei’s *D* statistic measures the separation of the treatment curves and can be interpreted as an estimate of the log treatment ratio comparing two groups of equal size [27]. The goodness of fit for the full model was calculated using Royston and Sauberbrei’s $R_{D}^{2}$. Finally, to assess the discrimination performance of individual predictors in the model, we calculated Royston and Sauberbrei’s *D* for the model after removing predictors from the model one at a time, adding them sequentially to the model, and as individual predictors (see Supplementary Table 1). It is important to note that, in non-proportional hazard models, $R_{D}^{2}$ is analogous to but not strictly interpretable as a measure of explained variance. However, it is still a useful index of determination and for comparing the relative contributions of the individual predictors in the model [28].

### Cluster analysis

As an alternative to using the model to generate treatment curves for hypothetical cases, we utilised cluster analysis to identify actual sub-populations within our sample potentially at greater risk of longer diagnostic-treatment intervals. K-medoid clustering [29] (partitioning around medoids—the point within a cluster where dissimilarity with all other points is a minimum) using Gower’s distance [30] to measure (dis) similarity was performed over the model covariates. Age and Stage at diagnosis were excluded from the cluster analysis on the grounds that they carried minimal prognostic information. The optimal number of clusters was determined using the Silhouette method. The clusters were then entered into a flexible parametric model (df = 3) with a time-dependent component (df = 2) to derive treatment curves for each cluster.

## Results

### Sample characteristics

**Fig. 1:**
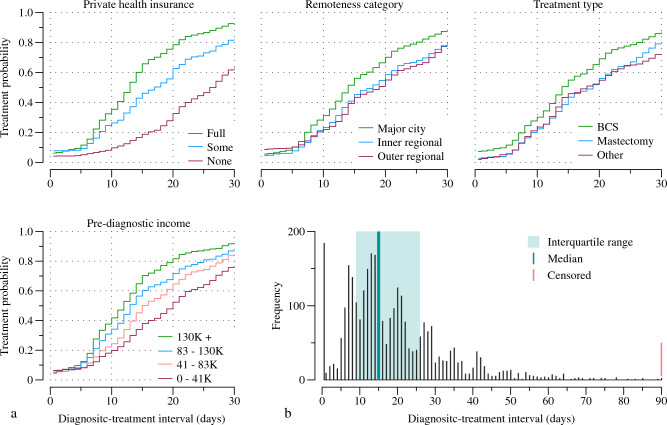
**a** Kaplan–Meier diagnostic-treatment interval estimates by covariate group, and **b** the distribution of diagnostic-treatment intervals. Note the *x*-axes of the Kaplan–Meier plots have been truncated for display purposes. Outer regional includes remote and very remote areas

The final model was run over a sample of 3216 participants. The mean age at diagnosis was 57.6 (SD 10.9) years and the median interval between breast cancer diagnosis and first treatment was 15 days, with an interquartile range of 9–26 days. The treatment curves varied by key factors (Fig. 1). Details regarding the sample breakdown and definitions for all variables retained in the final model and candidate variables can be found in Tables 1 and 2, respectively.

**Table 1:** Sample characteristics for all covariates retained in the final multivariable model, and explicit treatment delay

Model covariates	*n* (%)	Description	Fpm (df = 3) - *p*
Age	3216	Age at diagnosis	0.001
Range 26–80			
Median 58			
Private health insurance*		Self report: *“Do you have any private health insurance?*	0.001
Full health insurance	2037 (63.3)		
DVA/some private health Insurance	334 (10.4)		
No private health insurance	845 (26.3)		
Remoteness category		Categorised using the ARIA + classification	0.001
Major city	1899 (59.0)		
Inner regional	780 (24.3)		
Outer regional and remote	537 (16.7)		
Treatment type		Extracted from clinician and hospital records	0.001
BCS	2181 (67.8)		
Mastectomy	877 (27.3)		
Other (e.g. radiation, chemotherapy)	158 (4.9)		
Pre-diagnostic income		Self-report: *“Prior to your diagnosis, what was your annual household income before tax?”*	0.001
$0–$41,199	930 (28.9)		
$41,200–$83,199	904 (28.1)		
$83,200–$129,999	580 (18.0)		
+ $130,000	457 (14.2)		
Unknown	345 (10.7)		
Stage at diagnosis		Clinical stage at diagnosis obtained 12 months after diagnosis from patient medical records	
Stage I	1562 (48.6)		
Stage II/III	1235 (38.4)		
Stage IV	363 (11.3)		
Unknown	56 (1.7)		
Explicit treatment delay		Self-report: “*Were there any personal or practical delays in having this {insert relevant treatment}?”*	0.001
Treatment delay	446 (13.9)		
No treatment delay	2769 (86.1)		

*The ‘DVA/some private health insurance’ category included cases with basic hospital cover only, extras cover only, and health insurance policies provided by the Department of Veterans Affairs, which offers a limited degree of private hospital cover. These policies may not have covered aspects of treatment for breast cancer. Moreover, Private health insurance grants access to private hospital treatment, rather than treatment in public hospitals which is available to all Australians for free but can involve waiting lists that are prioritised according to need

**Table 2:** Additional candidate variables explored but not retained in the final multivariable model

Covariates	*n* (%)	Description	Fpm (df = 3) - p
Highest level of education		Self-report: *“What is the level of highest education you have completed?”*	0.001
Bachelor’s or higher	703 (21.9)		
Certificate or diploma	1259 (39.1)		
High School or less	1254 (39.0)		
Born in Australia		Self-report: *“Were you born in Australia?”*	0.001
Yes	2518 (78.3%)		
No	698 (21.7%)		
Aboriginal and Torres Straight Islander		Collected from Queensland	0.038
Yes	3169 (98.5)	Cancer Register records and	
No	47 (1.5)	Crossed check with self-report	
Family history of breast cancer		Self-report: *“Do any of your first-degree relatives {} have now or in the past had breast cancer?”*	0.05
Yes	703 (21.9)		
No	2431 (75.6)		
Don’t know	82 (2.5)		
Drive		Self-report: “Do you drive or have access to a car?”	0.001
Yes	2985 (92.9)		
No	229 (7.1)		
Miss	2 (0.1)		
Employed pre-diagnosis		Self-report: *“After your diagnosis, what was your annual household income before tax?”*	0.001
Full-time	1002 (31.2)		
Part-time/casual	891 (27.7)		
Home duties/carer/not working	339 (10.5)		
Retired	984 (30.6)		
Detection: Symptom v. screen		Self-report: *“How was your breast cancer first detected?”*	0.36
Symptom	1597 (49.7)		
Screen	1619 (50.3)		
Tumour grade		Histological tumour grade. Obtained 12 months after diagnosis from patient medical records	0.05
Low	599		
Intermediate	1541		
High grade	1032		
Unknown	44		

Associations reflect univariable analyses

Coefficient estimates from the final flexible parametric model are presented in Table 3. Private health insurance and remoteness category were modelled as time-dependent (TD) effects. Adding time-dependencies to the model increases the model’s complexity and makes interpreting the time-dependent coefficients (treatment ratios) difficult. This is because the time-dependent treatment ratios, by definition, vary over the interval. Additionally, when multiple time-dependencies are added, the treatment ratio of time-dependent covariates depends on the levels of the *other* time-dependent covariates in the model. Here we report treatment ratios (TR) at two timepoints—15- and 30-day intervals—where the treatment ratios for private health insurance and remoteness category have been calculated at the most common level of the other time-varying covariate—living in a major city and full private health insurance, respectively. Treatment ratios calculated across the other levels of the time-varying covariates showed only minimal differences. Treatment ratios over the full distribution for private health insurance and remoteness category are reported in Supplementary Fig. 1. To better highlight differences amongst the covariates, we refer to the treatment probabilities at discrete timepoints (see Table 4).

**Table 3:** Multivariable flexible parametric model (df = 3) with TD (df = 2)

Predictor	*n *(%)	TR 15 days (95% CI)	TR 30 days (95% CI)	*p *value
Age at diagnosis	3216	0.99	0.99 (0.99, 0.99)	0.001
Private health insurance (TD)				< 0.001
Full insurance	2037 (63.3)	1.00	1.00	
Some private insurance	334 (10.4)	0.66 (0.58, 0.74)	0.67 (0.56, 0.80)	*
No private insurance	845 (26.3)	0.31 (0.28, 0.35)	0.57 (0.50, 0.64)	*
Remoteness category (TD)				< 0.001
Major city	1899 (59.0)	1	1	
Inner regional	780 (24.3)	0.81 (0.74, 0.88)	0.94 (0.83, 1.07)	*
Outer regional and remote	537 (16.7)	0.71 (0.64, 0.79)	0.84 (0.73, 0.97)	*
Treatment type				0.001
BCS	2181 (67.8)	1.00	1.00	
Mastectomy	877 (27.3)	0.84 (0.77, 0.91)	0.84 (0.77, 0.91)	*
Other	158 (4.9)	0.60 (0.50, 0.71)	0.60 (0.50, 0.71)	*
Pre-diagnostic income				0.001
0–$41,199	930 (28.9)	1.00	1.00	
$41,200–$83,199	904 (28.1)	1.07 (0.97, 1.18)	1.07 (0.97, 1.18)	
$83,200, $129,999	580 (18.0)	1.16 (1.04, 1.30)	1.16 (1.04, 1.30)	*
$130,000+	457 (14.2)	1.26 (1.11, 1.44)	1.26 (1.11, 1.44)	*
Unknown	345 (10.7)	1.09 (0.96, 1.24)	1.09 (0.96, 1.24)	
Stage at diagnosis				
Stage I	1562 (48.6)	1.00	1.00	0.05
Stage IIA/IIB	1235 (38.4)	1.07 (0.99, 1.16)	1.07 (0.99, 1.16)	
Stage IIIA/IIIB/IV	363 (11.3)	1.17 (1.04, 1.32)	1.17 (1.04, 1.32)	
Unknown	56 (1.7)	1.07 (0.82, 1.42)	1.07 (0.82, 1.42)	

*p < 0.05

**Table 4:** Proportion treated by select diagnostic-treatment intervals, and median diagnostic-treatment interval (rounded to nearest day) by covariate

Covariate	Proportion treated		Median diagnostic-treatment interval (days)
	15 days (sample median)	30 days	
Total	0.47	0.83	15
Private health insurance			
Full private insurance	0.58	0.92	13
Some private insurance	0.44	0.82	16
No private insurance	0.20	0.63	25
Pre-diagnostic income			
0–$41,599	0.44	0.81	17
$41,600–$83,199	0.46	0.83	16
$83,200, $129,999	0.49	0.85	15
$130,000+	0.51	0.87	15
Remoteness category			
Major city	0.51	0.86	15
Inner regional	0.43	0.81	17
Outer regional and remote	0.39	0.78	18
Treatment type			
BCS	0.49	0.85	15
Mastectomy	0.44	0.81	17
Other	0.35	0.72	20

Estimates have been calculated from direct adjusted treatment curves

**Fig. 2:**
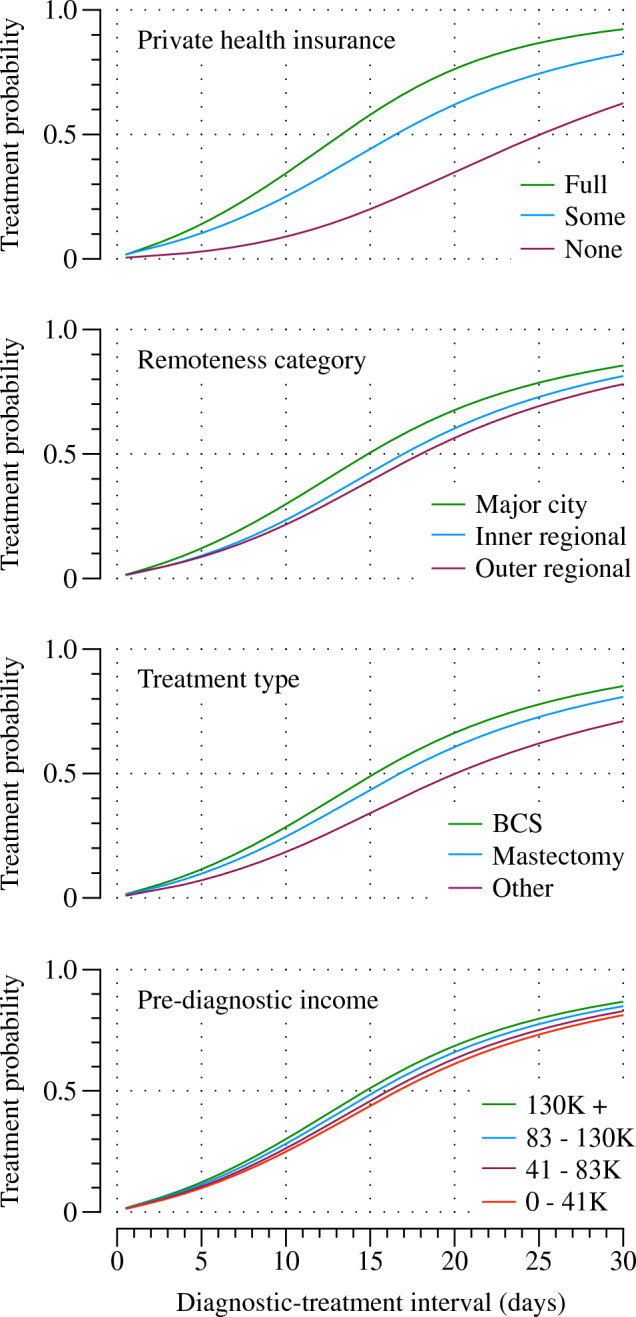
Direct adjusted treatment curves derived from the flexible parametric model (df = 3, TD df = 2), by covariate. Note the *x*-axes of the treatment curves have been truncated for display purposes

Broadly speaking, the final model shows that, on average, wait-times for first treatment following breast cancer diagnosis were *shorter* for those with full private health insurance, those living in a major city, those with higher pre-diagnostic income, and those whose initial treatment was BCS. Note that we observed a significant but small association between tumour grade and the diagnostic-treatment interval in the multivariable model. However, we chose to exclude tumour grade from the final model on the grounds that the effect was not clinically relevant—differences of 1 day between grade 3 and grade 1—and added additional complexity to the model by adding an additional time dependency. Stage at diagnosis was included in the final model to control for potential confounding, particularly of treatment type, and given its prognostic importance in other contexts. Note though that it was not associated with the outcome here and produced only minimal changes to the coefficients of the other variables. To better capture the difference between groups, particularly for time-dependent effects, the treatment probabilities for each group at 15 (sample median) and 30 days after diagnosis—a loose treatment guideline [22]—are shown in Table 4, along with the predicted treatment curves in Fig. 2. Treatment probabilities and curves for the levels of each predictor are derived from directly adjusted treatment curves. This approach involves estimating a treatment curve for every combination of covariates and averaging them according to weights defined by the frequency of the covariate pattern. The resulting estimates reflect the probability of having received treatment at a given point in time, if each group had the distribution of covariates in the study sample. The final column of Table 4 shows the estimated median diagnostic-treatment interval by covariate level and gives a sense of the differences in wait-times for treatment between the groups.

The final model explained approximately 13.8% of the variance in diagnostic-treatment intervals, $R_{D}^{2} = 0.138 \left( {95\% {\text{CI}}\;0.118, \;0.158} \right)$, with a Royston and Sauerbrei’s *D* of 0.82, demonstrating low to moderate discrimination performance of the model. Nearly all the prognostic information is carried by private health insurance, $R_{D}^{2} = 0.211 \left( {95\% {\text{CI}}\;0.179, 0.243} \right)$ as a single predictor model. Separately, the remaining predictors, age at diagnosis, pre-diagnostic income, and treatment type together accounted for approximately 4.9% of the variance, $R_{D}^{2} = 0.049 \left( {95\% {\text{CI}}\;0.037, \;0.061} \right)$ (Supplementary Table 1). The unusual pattern whereby $R_{D}^{2}$ *decreased* with the addition of significant predictors in the model, to the extent that private health insurance alone appears to account for more variance than the final model, is likely related to a shifting baseline function, from which $R_{D}^{2}$ is derived.

Much of the variance in the diagnostic-treatment interval remains unexplained, however, some of this variation may be unrelated to the specific characteristics and circumstances of the women in the study. That is, some of the variation may reflect the randomness of daily routine and situation; for example, some patients reported delays to treatment due to the major floods in Brisbane in 2011, factors that are of less interest in a model aimed at capturing systematic differences. We extended the model to include ‘explicit treatment delay’ to potentially capture and quantify some of this additional variance. Those who explicitly reported experiencing personal or practical delays to their first breast cancer treatment experienced longer intervals between diagnosis and first treatment, on average, than those who did not report a delay, *p* < 0.001. Inclusions of explicit treatment delay into the model improved discrimination performance (Sauerbrei’s *D* = 1.03) and explained an additional 6.5% of the variance in diagnostic-treatment intervals, $R_{D}^{2} = 0.202 \left( {95\% {\text{CI}}\,0.182,\, 0.224} \right)$.

### Cluster analysis

**Fig. 3:**
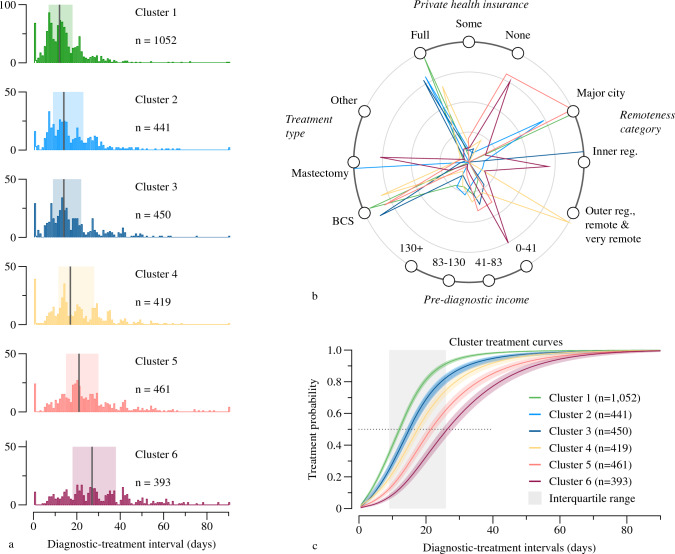
**a** Distribution of diagnostic-treatment intervals by cluster. **b** Radar plot showing the characteristics of women within each cluster. The concentric circles reflect the percentage of women in each cluster with a given characteristic, where the centre reflects 0% and expands outwards to 100%. Jitter (2°) has been added to the clusters to help differentiate them. **c** Directly adjusted treatment curves derived from the flexible parametric model (df = 3, TD df = 2), by cluster. The shaded bands reflect the 95% confidence intervals

The Silhouette method identified five optimal clusters. Figure 3 shows (a) the distribution of diagnostic-treatment intervals for all clusters, (b) the covariate patterns for each cluster, and (c) the adjusted treatment curves for each cluster. Importantly, the clusters are differentiated along the diagnostic-treatment interval. The median diagnostic-treatment interval increases across the clusters, where the cluster 1 median is 12 days between diagnosis and first treatment compared to 27 days for cluster 6. At the sample median of 15 days, 64% and 19% of women in cluster 1 and cluster 6 had received their first treatment, respectively (see Fig. 3). At 30 days post-diagnosis, only 6% of women in cluster 1 had *not* received their first treatment, whilst 40% of women in cluster 6 were still waiting for their first treatment.

Clusters 5 and 6—the clusters with the longest wait times for first treatment—are dominated by women who had no private health insurance, and a minority that had partial insurance. Remoteness also characterised the clusters: amongst Clusters 1–3, in which most women had full insurance, treatment times increased as the predominant remoteness category changed from major city to outer regional and remote. Clusters in which most women had less physical access to health services (that is, lived outside major cities) but had greater financial access in terms of full health insurance tended to have faster treatment times than women who lived nearer services but did not have full health insurance. The clusters with high proportions of women with full health insurance had approximately equal numbers from each of the categories for pre-diagnostic income. Women in the clusters without full private health insurance also tended to be in the lowest or second to lowest categories for pre-diagnostic income.

## Discussion

A central tenet of breast cancer management and care is early detection and diagnosis to expedite treatment, where timely treatment is associated with improved breast cancer outcomes [8, 31–34]. Delays to the initiation of treatment can lead to poorer prognosis if disease is allowed to advance [3, 4, 11, 31, 34]. Whilst prevention where possible is ideal, timely treatment following diagnosis offers the best chance of reducing breast cancer burden. In the current study, we examined how long women waited for their first treatment following a breast cancer diagnosis, and the factors that predicted their wait-time.

That the median interval between breast cancer diagnosis and first treatment was 15 days, with 84% of the cohort receiving treatment within 30 days of diagnosis, is encouraging from the perspective of breast cancer biology. The estimated breast tumour doubling times vary widely, between 45 and 260 days [4]. Guidelines for an acceptable diagnostic-treatment interval length are scarce. However, it has been suggested that diagnostic-treatment intervals of 30 days or less are unlikely to negatively affect survival [22], and Cancer Australia [35] recommends surgery occurs within 30 days of diagnosis for those not receiving neoadjuvant therapy. We found that approximately one in six women (16%) waited longer than 30 days post-diagnosis for treatment. Bleicher [4] suggests that surgeries should proceed within 90 days of a breast cancer diagnosis, where diagnostic-treatment intervals greater than 90 days have been associated with poorer survival [36]. It is therefore encouraging that only 13 out of 3216 women in our study waited 90 days or longer. Of course, the urgency of *treatment* may depend heavily on the previous detection and diagnostic pathway.

We identified several socioeconomic factors associated with the breast cancer diagnostic-treatment interval. The predictor with the largest effect size was private health insurance. On average, those with full private health insurance received first-treatment nearly *2 weeks* sooner than those with no private health insurance. Moreover, more than 1 in 3 women (37%) with no private health insurance waited longer than 30 days for their first treatment following diagnosis. Note that the effect of health insurance was time-dependent though, and the benefits associated with full private health insurance relative to no private health insurance diminished as the length of the diagnostic-treatment interval increased. Other predictors of treatment wait time included pre-diagnostic income and remoteness, where those earning more and residing in major cities tended to experience shorter wait-times between diagnosis and first treatment. Though small, note that the effect of pre-diagnostic income is independent of private health insurance, indicating that there is advantage attached to income beyond granting access to full private health insurance. Possibly income affords greater flexibility when it comes to the scheduling of treatments, and or the ability to organise existing commitments to prioritise urgent health care. Additionally, we observed an effect of treatment type, whereby patients waited less time for BCS relative to mastectomy and other treatment types, after adjusting for stage at diagnosis. Note that only a small percentage (3.2%) of women in the study cohort received pre-operative neoadjuvant chemotherapy (NAC), where the proportion of patients receiving NAC in Australia has steadily increased over the last decade towards a target of 20% [37, 38]. Importantly, NAC can extend the interval to treatment due to increased multidisciplinary discussion of treatment planning and management [39], suggesting that the interval differences observed here for treatment type may underestimate current differences. Overall, much of the variation in the diagnostic-treatment interval remained unexplained though, even after accounting for a source of variance unrelated to the inherent characteristics of the women in the study—explicit treatment delay. Finally, the underrepresentation of advanced disease in our sample is worth noting. Such bias is difficult to avoid in the study of serious disease, and it is difficult to know what consequence it should have for our interpretation of the results. Given that private health insurance was negatively associated with disease advancement in our sample (*p* < 0.001), it’s possible that non-responders were *less* likely to have full private health insurance, and consequently may indicate an *underestimation* of the true diagnostic-treatment intervals differences reported here.

Whilst many of the differences in diagnostic-treatment intervals we observed were small, it is important to consider that some of these same factors have also been linked to screening and diagnostic delay, and accumulation of delay at multiple stages along the diagnostic and treatment pathway may harm outcomes. Moreover, the cluster analysis suggests that there are dependencies between these predictors in the population, and small effects across multiple factors accumulate, potentially resulting in poorer breast cancer outcomes for those individuals. Although these data are approximately 10 years old now, and may reflect dated treatment pathways, they provide unique insights that are otherwise not possible with more recent, routinely collected data from cancer registers—they do not routinely collect the breadth of socioeconomic data we have reported here. Though breast cancer treatment pathways may have changed, increasing costs in healthcare and growing gaps in health outcomes across socioeconomic groups in Australia [40], suggest that the effects reported here likely persist, and highlights the need for more contemporary data. Additionally, the consequences of variation in cancer treatment and care can take years to manifest and identify. The data reported here are relevant to understanding current inequalities in breast cancer outcomes in Queensland, Australia, and potentially, more broadly wherever socio-economic gradients resemble those in this study. Indeed, Australia boasts one of the best health care systems in the world [41], the patterns of treatment inequality reported here may even be exaggerated in other regions with less equitable and efficient healthcare systems. Identifying effective strategies to reduce the disparity in wait-times for breast cancer treatment faced by socioeconomically disadvantaged women should remain a priority.

### Supplementary Information

Below is the link to the electronic supplementary material.

Supplementary file1 (DOCX 68 KB)

## Data Availability

The data supporting this research are exclusively accessible through a formal Data Sharing Agreement, which can be initiated by contacting the corresponding author, and may be subject to eligibility criteria. The agreement outlines the terms and conditions governing data usage, security, and ethical considerations.
