# Supplementary material for: Individual and area level factors associated with the breast cancer diagnostic-treatment interval in Queensland, Australia
Source: Breast Cancer Res Treat. 2023 Nov 6;203(3):575–86. doi: 10.1007/s10549-023-07134-4 (PMC10805972; doi:10.1007/s10549-023-07134-4)
Supplement: Supplementary file 1 — Supplementary file1 (DOCX 68 KB) [file 10549_2023_7134_MOESM1_ESM.docx]

**Supplementary**

Supplementary Table 1. Discrimination indices and ${R_{D}}^{2}$ for all model predictors, with each predictor removed, added sequentially, and individually.

|  | With predictor removed | | Sequentially added | | Individually | |
| --- | --- | --- | --- | --- | --- | --- |
| Predictor | R&S’s D | ${R_{D}}^{2}$ | R&S’s D | ${R_{D}}^{2}$ | R&S’s D | ${R_{D}}^{2}$ |
| Model | 0.828 | 0.137 | - | - | - | - |
| Private health insurance | 0.475 | 0.047 | 1.058 | 0.211 | 1.058 | 0.211 |
| Pre-diagnostic income | 0.802 | 0.133 | 0.690 | 0.102 | 0.374 | 0.032 |
| Remoteness Category | 0.742 | 0.116 | 0.780 | 0.127 | 0.293 | 0.020 |
| Treatment type | 0.770 | 0.124 | 0.799 | 0.132 | 0.282 | 0.019 |
| Age at diagnosis | 0.799 | 0.132 | 0.816 | 0.137 | 0.003 | 0.119 |

**Supp Fig.1** Treatment ratios for the time-varying covariates – private health insurance and remoteness category. The reference categories for private health insurance and remoteness category are full private health insurance and living in a major city, respectively. The shaded bands around the treatment ratios reflect the 95% confidence interval. The vertical grey bands reflect the sample inter-quartile range. The treatment ratios have been calculated at the most common category of the other covariates e.g., the treatment ratios for private health insurance are calculated for those living in a major city.
